# Rigid Schiff Base Complex Supermolecular Aggregates as a High-Performance pH Probe: Study on the Enhancement of the Aggregation-Caused Quenching (ACQ) Effect via the Substitution of Halogen Atoms

**DOI:** 10.3390/ijms23116259

**Published:** 2022-06-02

**Authors:** Tianyu Li, Haijun Pang, Qiong Wu, Meifen Huang, Jiajun Xu, Liping Zheng, Baoling Wang, Yongfeng Qiao

**Affiliations:** 1 Department of Chemical Science and Technology, Kunming University, Kunming 650214, China; lty176666@gmail.com (T.L.); hmf668648@gmail.com (M.H.); xjj1999122@gmail.com (J.X.); zhengliping958@gmail.com (L.Z.); wangbaolingkmu@gmail.com (B.W.); 2 College of Physics Science and Technology, Kunming University, Kunming 650214, China; 3 The School of Material Science and Chemical Engineering, Harbin University of Science and Technology, Harbin 150040, China; panghj116@163.com; 4 Yunnan Engineering Technology Research Center for Plastic Films, Kunming University, Kunming 650214, China

**Keywords:** rigid Schiff base complex, halogen bond, supermolecular aggregates, fluorescence enhancement, DFT studies

## Abstract

Optical signals of pH probes mainly driven from the formation or rupture of covalent bonds, whereas the changes in covalent bonds usually require higher chemical driving forces, resulting in limited sensitivity and reversibility of the probes. The exploration of high-performance pH probes has been a subject of intense investigation. Herein, a new pH probe has been developed, with optical property investigation suggesting the probe has excellent signal-to-noise ratio, and fluorescence intensity shows exponential growth, combined with a visible color change, as pH increased from 5.1 to 6.0; Moreover, the probe has outstanding stability and reversibility, with more than 90% of the initial signal intensity remaining after 30 cycles. In order to better understand the special fluorescence behavior of the reported probe, the non-halogenated isomer is introduced for comparison, combined with the results of structural analysis, quantitative calculation and optical experiments, and the possible mechanism of the special supramolecular aggregation-caused quenching effect induced by the halogen atom is discussed.

## 1. Introduction

The concentration of hydrogen ions (pH value) in solutions is not only an important analytical index in chemical synthesis, agricultural production and water detection, but also plays an important role in many physiological processes, such as proliferation, apoptosis, ions transportation, endocytosis and muscle contraction [1,2,3,4]. Therefore, the availability of rapid and convenient methods for accurate detection of pH value is crucial to many scientific fields [5]. A pH fluorescence sensor is a kind of detecting device based on spectroscopy measurement, which can express the pH information of the detected objects via fluorescence signal and has many advantages including being a simple device, and allowing on-line analysis and real-time monitoring [6,7]. In recent years, with the rapid development of emerging fields such as bioengineering, environmental science and fine chemical industry, the requirement for accuracy, precision and stability of pH fluorescence sensors is becoming ever more demanding [8].

Up to the present, although a variety of molecular-based pH sensors have been developed, most of which are built up from fixed covalent conjugated architectures [9,10,11,12,13], the formation and rupture of covalent bonds require higher chemical driving forces. Small changes in the external environment have little effect on the conversion of covalent bonds, which usually leads to limited sensitivity and reversibility. Therefore, the design and synthesis of high-performance probes to meet the demand of complex applications is still a big challenge.

Supramolecular assembly is a type of molecular aggregate assembled by intermolecular recognition, for which physicochemical properties can be regulated via the adjustment of aggregation states, rather than changing the intramolecular structure [14,15,16]. Compared with covalent compounds, the structural properties of supramolecular assemblies can be changed at much lower chemical and physical driving forces. Hence, their response is more sensitive to external stimuli [17,18]. The driving forces for supramolecular assemblies are commonly attributed to intermolecular interactions, such as π–π stacking, hydrogen bonding, halogen bonding and electrostatic interactions, which can be greatly changed with the variation of the H^+^ environment [17,18,19,20,21]. If the intermolecular interactions can take advantage of H^+^ as a driving force to construct specific supramolecular aggregates, a new type of pH probe based on non-covalent bonds can be achieved. For example, if the supermolecular assembly induced by H^+^ is ACQ (aggregation-caused quenching)-active [22,23,24], the probe molecules can exhibit strong fluorescence emission in their isolate form, and the intensity could be quenched after polymerization; Likewise, if the supermolecular assembly is AIE (aggregation-induced emission)-active [25,26,27,28,29], the opposite emission process reflects the change in external H^+^. The realization of a supramolecular probe depends on two requisites: (1) The probe requires adequate stability at a broader pH range, where intramolecular structure will not be changed with the variation of H^+^; (2) The supramolecular aggregates are sensitive to the external H^+^ environment and the change in pH can significantly affect the aggregate states. Therefore, the establishment of an ideal supermolecular model for pH probes remains a challenge.

Salen is an important branch of Schiff base compounds with diverse structures and properties, in which electron density and the conjugated system can be easily regulated by the incorporation of different salicylate aldehydes and diamines [30,31]. Their specific fluorescence behaviors have been widely used as fluorescence probes in the field of ion recognition [32,33,34,35,36,37]. However, due to the small change in fluorescence intensity of Salen-type compounds in the different pH environments, the indistinguishable signal makes it difficult to be applied as a pH chemosensor. Therefore, the progress of Salen-type pH sensor is very limited, and the improvement of the signal intensity and reversibility of a Salen-type probe has become a major bottleneck in this field. On the other hand, Salen is a class of important molecules in material science that have been intensively studied for decades; Introducing this class of molecules as a research model and combining the experimental and theoretical results, their special phenomena can be explained in more detail at a molecular and electronic level [38,39,40].

In this study, we chose pentadentate halogenated Salen (saldmpn) as the chelate ligand, compared with traditional quasi-planar Salen complexes, saldmpns tend to form rigid chelating structures with transition metals, which have a wide range of pH stability and can provide an ideal research model for supramolecular interactions. Through the systematic screening of transition metals, a new nickel (II) supramolecular aggregate Ni-χ-L (Ni^II^(3,5-Cl-saldmpn)) was isolated. Fluorescence experiments showed that Ni-χ-L exhibits a fast response to different pHs and the emission intensity at 525 nm has noticeably increased 15 times, as pH changed from 5.1 to 6.0. Such high signal-to-noise ratios (SNR) can effectively avoid the adverse effects of self-luminescence and ambient light, meanwhile, the probe exhibited excellent linearity, stability and reversibility.

## 2. Results

### 2.1. Photophysical Properties

**Figure 1:**
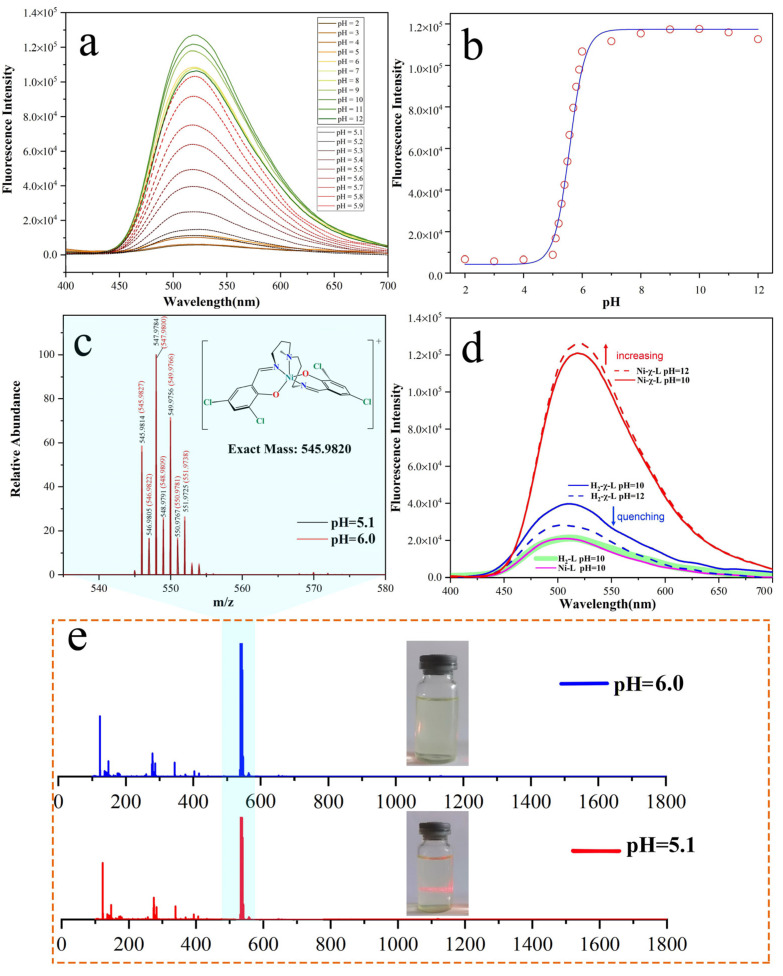
(**a**) Fluorescence spectra of Ni-χ-L in different pH solutions; (**b**) plot of maximum intensity vs. pH of Ni-χ-L; (**c**,**e**) amplified and full ESI-MS spectra of Ni-χ-L at pH 5.1 and 6; (**d**) the comparison of FL at pH higher than 10.

To explore pH-dependence optical properties of the synthesized rigid Schiff base complex Ni-χ-L, the fluorescence titration at different pHs in ethanol solution was carried out. As shown in Figure 1a and Figure S1, the maximum emission peak positions of its solution remain unchanged, with the pH changes from approximately 2 to 12, and the variation in emission intensity at pH 2~5 was small and, overall, showed fluorescence quenching. Unexpectedly, the fluorescence intensity increased 15-fold with the variation of one pH unit, and showed very good linearity within this range (Figure 1b). This anomalous optical behavior has aroused our great interest. It is known that the coordination complex has stabilities in a certain range of pHs [41], especially the pH value around its synthesis environment (Ni-χ-L: pH = 4.8); the variation of one pH unit causing such a significant difference in emission intensity is a rare optical phenomenon. To better understand this observation, high-resolution electrospray ionization mass spectrometry was firstly performed, as shown in Figure 1c and Figure S2, from which we can see that the ion peaks of these two samples at pH 5.1 and 6.0 are completely coincided with each other, suggesting the intermolecular composition of these two samples is exactly the same. In order to further verify the stability of Ni-χ-L, we compared changing trends in the photoluminescence (PL) intensity of Ni-χ-L and its ligand H_2_-χ-L (3:5-Cl-saldmpn = N,N’-( 3,3′-dipropyhnethylamine) bis (3,5-chlorosalicylidene)) in a higher pH range, from 10 to 12 (Figure 1d); the fluorescence intensity of Ni-χ-L is increased, while the ligand shows quenching at the same pH range. These results both show the fluorescence behavior of Ni-χ-L is different from that of the halogenated ligand H_2_-χ-L and also confirms the good stability of Ni-χ-L in a strong alkaline environment.

**Figure 2:**
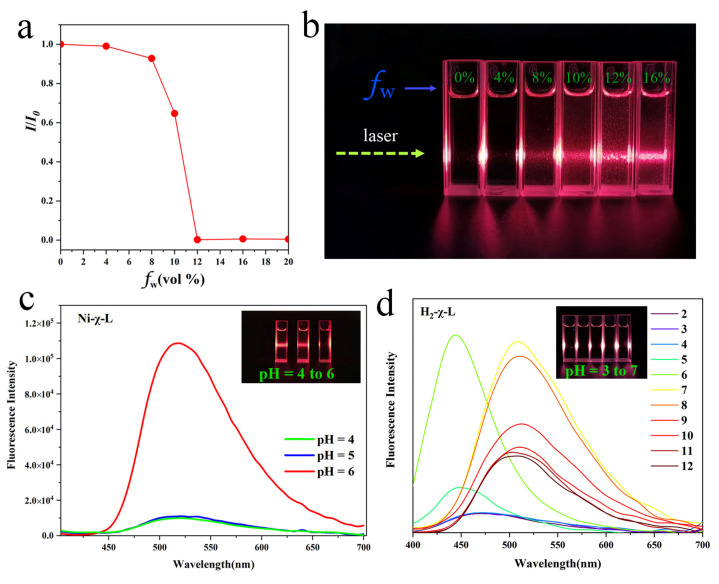
(**a**) Tyndall test of Ni-χ-L at various water fraction (concentration: 400 μM), the excitation wavelength was 360 nm (**a**), plot of (I/I_0_) of Ni-χ-L versus the water fraction from 0% to 20% (**b**), fluorescence spectra as well as the Tyndall effect of (**c**) Ni-χ-L and (**d**) H_2_-χ-L at the key pH value. (c = 400 μmol/L, EtOH/water = 20/1, *v*/*v*, λ_ex_ = 360 nm).

The state of molecular aggregations can be regulated by different intermolecular interactions and resulting different optical behaviors [29,30]. Thus, we speculate this phenomenon is related to the aggregation states of Ni-χ-L, and studied the potential aggregated states via characteristic photophysical behavior in varying fractions of a poor solvent and different pH values [42]. As shown in Figure 2a and Figure S3, the PL intensity was nearly unchanged before the content of water is less than 8%; however, when water content reached 10%, an obvious PL intensity decrease was observed and the intensity was almost completely quenched as the water fraction increased to 12%. The remaining PL intensity was only 1% compared to that of the pure ethanol solution. The observed PL changes were generally in good agreement with the brightness of the Tyndall phenomena and Mie effect formed light path (Figure 2b), that is, halogenated Schiff base complexes molecular aggregation formed non-radiative transitions suggesting typical ACQ behavior. The same comparison was also performed in different pH environments (Figure 2c). Unsurprisingly, the observation is similar to that of the poor solvent. The solution of Ni-χ-L also presents an obvious Tyndall phenomenon and Mie effect at a lower pH value, revealing the aggregation state of Ni-χ-L can be generated by increasing the concentration of H^+^.

Emission spectra of H_2_-χ-L was measured in the pH range of 2.0 to 12. With the pH increased from 2.0 to 6.0, the peak at 466 nm increased and appeared blue-shifted to 442 nm. However, as the pH was increased further, a new peak appeared at 507 nm at pH 7.0, this observation is similar to previous studies, which can be attributed to the deprotonation process of the Schiff base compound. Different to Ni-χ-L, the intensity decreased with the increasing pH (from 7.0 to 12.0), suggesting the concentration of the deprotonated form increases, leading to the quenching behavior. Meanwhile, the Tyndall effect also showed no difference between the samples, suggesting the variation of FL (fluorescence) of H_2_-χ-L mainly originates from a change in intramolecular structures rather than the formation of the aggregation state.

According to the above experimental results and Figure S4, Ni-χ-L switches between isolated monomers and supramolecular aggregates with the variation of pH values, resulting in different fluorescence behaviors, that is, Ni-χ-L exhibits a strong fluorescence emission in the isolated form (pH > 6.0), and the intensity could be quenched after polymerization (pH < 5.1).

**Figure 3:**
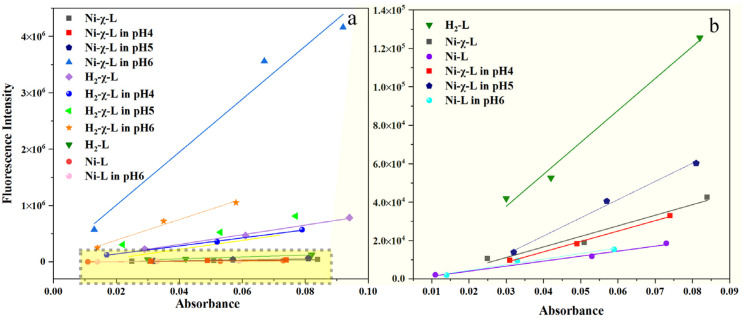
(**a**) Plots of integrated fluorescence intensity of Ni-χ-L in pH = 6.0, H_2_-χ-L, H_2_-L, Ni-χ-L and Ni-L and quinine sulfate (referenced dye) as a function of optical absorbance at 360 nm; (**b**) right figure showed the amplified lines below 1.4 × 10^5^. (c = 400 μmol/L, EtOH/water = 20/1, *v*/*v*, λ_ex_ = 360 nm).

To further validate the origination of the strong emission above pH 5.1, as well as the influence of halogenated ligands on the optical behavior of the complex, the quantum yield of Ni-χ-L, H_2_-χ-L, Ni-L (Ni^II^(3,5-Cl-saldmpn)) and H_2_L (saldmpn = N, N’-( 3,3′-dipropyhnethylamine) bis (salicylideneiminato)) at important feature points were measured by using a relative approach. As is shown in Figure 3a,b, without changing pH, the quantum yield of Ni-χ-L in the synthesis solution has been calculated to be 0.1%, which is obviously lower than that of its ligand H_2_-χ-L 1.6%. Reducing by 0.8 pH unit to pH 4.0, a slight QY (0.1%) reduction can be observed. Combined with the Tyndall effect, this phenomenon could be associated with a further aggregation process [43]. The apparent increase in QY of Ni-χ-L occurred after pH > 5.1 (0.2%), which rapidly reached 8.6% at pH = 6. This observation is in accordance with the formation of isolated ACQ-active molecules. Meanwhile, we compared the quantum yields of non-halogenated isomers, with results showing that QY of the non-halogenated isomers at pH 4.8 and 6 are basically the same (0.05% and 0.06%, respectively), suggesting the interaction between rigid Schiff base complexes can be significantly enhanced via the substitution of halogen atoms, resulting in a fluorescence quenching effect (ACQ).

**Figure 4:**
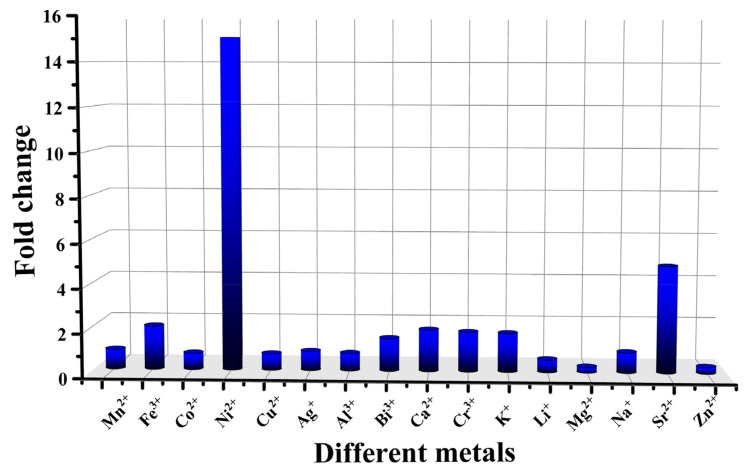
Fold changes of different complexes (Mn^2+^, Fe^3+^, Co^2+^, Ni^2+^, Cu^2+^, Ag^+^, Al^3+^, Bi^3+^, Ca^2+^, Cr^2+^, K^+^, Li^+^, Mg^2+^, Na^+^, Sr^2+^ and Zn^2+^) in ethanol solution changed from pH 5.1 to 6.0.

In order to evaluate the differences in the enhancement effect of halogenated Schiff base ligands on different metals at a pH range of 5.1 to 6.0, we compared the fold changes of a series of complexes based on saldmpn^2-^ (Mn^2+^, Fe^3+^, Ni^2+^, Cu^2+^, Ag^+^, Al^3+^, Bi^3+^, Ca^2+^, Cr^3+^, K^+^, Li^+^, Mg^2+^, Na^+^, Zn^2+^ and Sr^2+^). The comparison results are shown in Figure 4. It can be seen that saldmpn-type complexes generally showed the same enhanced trend at a change of pH 5.1 to 6.0, whereas, the fold changes of fluorescence intensity of Ni complexes is obviously higher than the other metal complexes. These results indicate that Ni-χ-L has the highest sensitivity and discrimination from pH 5.1 to 6.0, which makes it an ideal candidate for a pH probe at this range.

**Figure 5:**
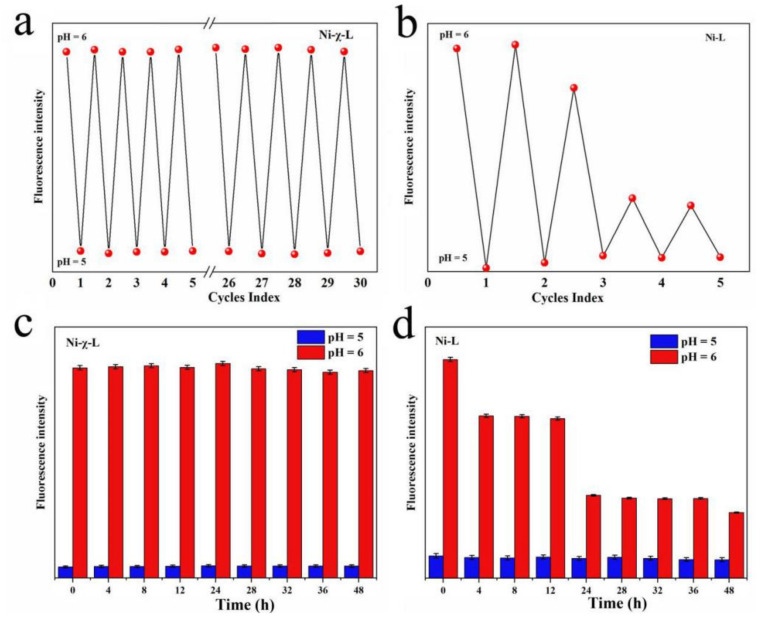
Reversibility and stability of Ni-χ-L and Ni-L: (**a**) reversibility of Ni-χ-L; (**b**) reversibility of Ni-L; (**c**) stability of Ni-χ-L; (**d**) stability of Ni-L. (c = 400 μmol/L, EtOH/water = 20/1, *v*/*v*, λ_ex_ = 360 nm).

Recognizing the sensitivity of the supermolecular aggregates and the monomers to pH, the reversibility of these two forms attracted our interest. It was found that the strong fluorescence emission of the solution can be quenched after the pH was adjusted down to 5.1 with the addition of HCl, and fluorescence intensity can be almost fully recovered via a pH adjusted above 6. Interestingly, the reaction can quickly respond and keep the signal intensity at more than 90% of the initial signal intensity after 30 cycles (Figure 5a). These results indicate that, unlike the previously reported covalent bond or strong interaction-based pH sensors, the halogen-bond-induced pH sensor features superior reversibility and conversion speed between different forms. Comparison experiments show that the reversibility of the non-halogenated complex Ni-L is obviously decreased (Figure 5b), which may be related to the poor stability of the non-halogenated complex. Stability is another important reference index for chemical sensors, and stability comparison test results are shown in Figure 5c,d. It can be seen that the fluorescence intensity of Ni-χ-L at these two pH values did not change significantly within 48 h, indicating that both of the isolated and aggregated forms exhibited good stability. However, under the same conditions, the fluorescence intensity of a non-halogenated isomer at pH = 6.0 dramatically changed after 12 h (Figure 5d), indicating the lower stability of the non-halogenated isomer.

### 2.2. Single-Crystal X-ray Diffraction Analyses

**Figure 6:**
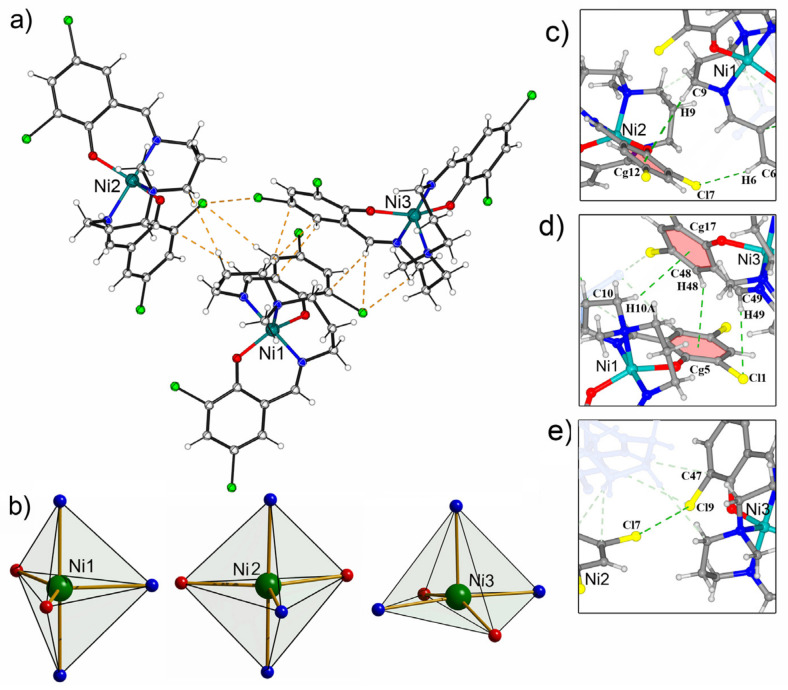
(**a**) Ball-and-stick representation of the asymmetric unit of the trimeric aggregation {[Ni^II^(3,5-Cl-saldmpn)]_3_}(1); (**b**) the coordination polyhedrons for Ni(1), Ni(2) and Ni(3); (**c**) the amplified region of interactions between Ni(1) and Ni(2): C6-H6···Cl7, D-H···A = 2.9996(11) Å; D···A = 3.8902(47) Å, ∠D-H···A = 156.652(283)°; C9-H9···Cg12 (Cg12: C36-C37-C38-C39-C40-C41): D-H···A = 3.3327(1) Å; D···A = 4.2896(58) Å, ∠D-H···A = 163.062(338)°; (**d**) the amplified region of interactions between Ni(1) and Ni(3): C49-H49···Cl1, D-H···A = 3.051(1) Å; D···A = 3.3953(53) Å, ∠D-H···A = 155.681(325)°; C48-48···Cg5 (Cg17: C1-C2-C3-C4-C5-C6): D-H···A = 2.9224(1) Å; D···A = 3.7894(52) Å, ∠D-H···A = 152.402(32)°; C10-H10A···Cg17 (Cg17: C43-C44-C45-C46-C47-C48): D-H···A = 3.076(1) Å; D···A = 3.8378(87) Å, ∠D-H···A = 134.768(47)°; (**e**) the amplified region of interactions between Ni(2) and Ni(3): C47-Cl9···Cl7, D-Cl···A = 3.2900(18) Å, ∠D-H···A = 165.335(178)°.

Although the fluorescence behavior of the reported compound comes from the intermolecular interactions in a solution environment, its solid-state aggregation is crucial to approve the proposed mechanism. Therefore, in order to obtain the structural information in the form of single crystal, we used anhydrous methanol as a solvent. Halogenated ligand H_2_saldmpn (H_2_-χ-L) reacts in a stoichiometric ratio of 1:1 with nickel chloride and brown crystals suitable for single-crystal diffraction were crystallized after a week. The detailed crystal data and structure refinement for Ni-χ-L is given in SI: Table S1. X-ray structural analyses reveals that Ni-χ-L crystallizes in the monoclinic system (space group P21/c). Unexpectedly, the asymmetry unit comprises three crystallographically independent, neutral Ni-χ-L (abbr. [Ni1], [Ni2] and [Ni3]) units with exactly the same composition (Figure 6), according to the search results in the Cambridge Crystallographic Database (CSD). Although some non-halogenated derivatives can be found in the database, all of the found structures exist in the form of isolated single molecules, and Ni-χ-L represents the first saldmpn-based supramolecular aggregates constructed from identical units, as shown in Figure 6a and Figure S6. Each saldmpn^2-^ acts as pentadentate ligands offering an {N_3_O_2_} coordination site for the central atom, that is, one amine (Nam) and two imino nitrogen atoms (Nim) and two phenolato oxygen atoms O*. The longest bond length around the metal center is the Ni-Nam bond ranging from 2.011 to 2.087 Å. The Ni-Nim bonds are a bit shorter (2.002–2.087 Å) and the Ni-O* bonds are the shortest (1.95–2.00 Å). These featured bond lengths of the studied Ni-χ-L are within the normal ranges of the previously reported saldmpn-type nickel complexes [44].

It is noteworthy that, as the central atoms adopt a pentacoordinate mode with saldmpn^2-^ ligands, there are two possibilities in its geometry: square-pyramidal (tetragonal cone SP) and trigonal-bipyramidal (TB). These two coordination geometries can be effectively determined by the Addison parameter τ [45]. From the calculation result, we can see that the τ values of the three Ni atoms are quite different (Figure 6b). Ni1 and Ni2 exhibit higher values (τ1 = 0.534 and τ2 0.619, respectively), while the τ value for Ni3 is much lower (τ3 = 0.333), which clearly indicates the geometry of Ni1 and Ni2 are close to TB, while the geometry of Ni3 belongs to SP.

**Figure 7:**
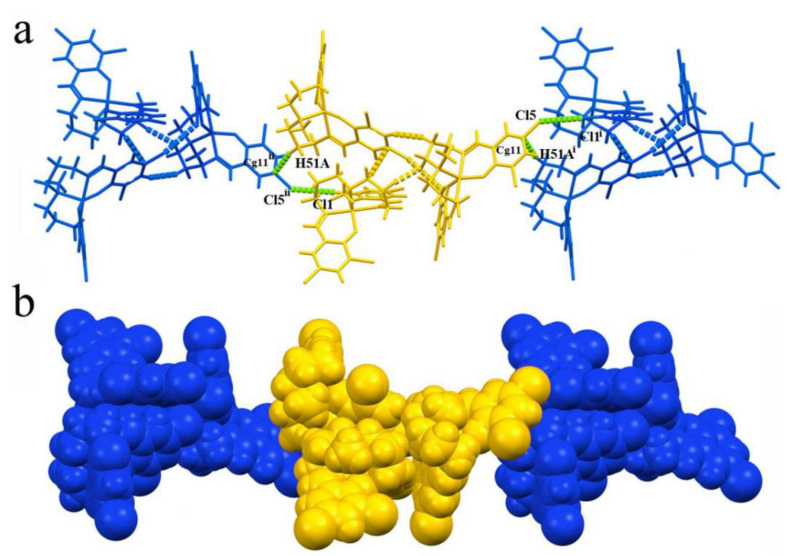
H-bonding and XB interactions formed 1D chain structure based on trimeric aggregations: (**a**) stick view; (**b**) space-filling view. C26-Cl5.

Weak intra- and intermolecular interaction analysis results show that, unlike common Salen-type compounds, no classic hydrogen bonds exist in the trimeric aggregation, but multiple intermolecular interactions are formed among the molecules, including a halogen bond, hydrogen bond and C-H···π. The interaction between [Ni1] and [Ni2] fragments are C-H···π and C-H···Cl; [Ni2] and [Ni3] is typical halogen bond C-Cl···Cl-governed interaction; and [Ni1] and [Ni3] are jointed multiple C-H···π and C-H···Cl interactions. Thanks to these synergetic effects of weak interaction, these three neutral molecules are linked to each other and form a unique trimeric supramolecular aggregate. Further, C-H···Cl interactions link the neighboring trimeric supramolecular aggregates into 1D chains, as shown in Figure 7.

## 3. Discussion

In order to better interpret the chemical reactivity, electrical structure and optical properties of the reported compound, theoretical calculations were performed by using the density functional theory (DFT) with the B3LYP functional levels for 6–31g(d,p) basis set [46,47]. It should be pointed out that, although there are three independent Ni complexes in the asymmetric unit, the experimental results suggested that the fluorescence intensity in pH 6 mainly comes from single molecule, so in this work, the atomic coordinates used for optimization are from the crystal structure of the Ni1 fragment. In addition, for the purpose of revealing the influence of the halogen atom on the fluorescence behavior, the non-halogenated isomer Ni-χ-L was introduced for comparison. As is shown in SI: Figure S7, the optimized geometry of the Ni1 fragment is in good agreement with the crystal data. The strong coincidence between the optimized structure and crystal structure indicates the excellent stability and rigidity of Ni-χ-L.

The electron cloud of Ni-χ-L at the HOMO level is distributed over the whole molecule, while the LUMO electron cloud is mainly concentrated in the introduced diphenylenamine part, indicating that there is a certain degree of charge transition. In addition, due to the introduction of the halogen atoms, the electron cloud on the Ni-χ-L-benzene ring conjugate surface has a larger distribution area, significantly improving the molecular conjugation; which increases the intermolecular interactions and tends to interact with the π plane of other nearby molecules, leading to the formation of more non-radiation paths. In contrast, the conjugate plane of the non-halogenated isomer is smaller and the rigidity of the whole molecule led to a more evident spatial effect and limited the potential path of non-radiation between adjacent molecules.

**Figure 8:**
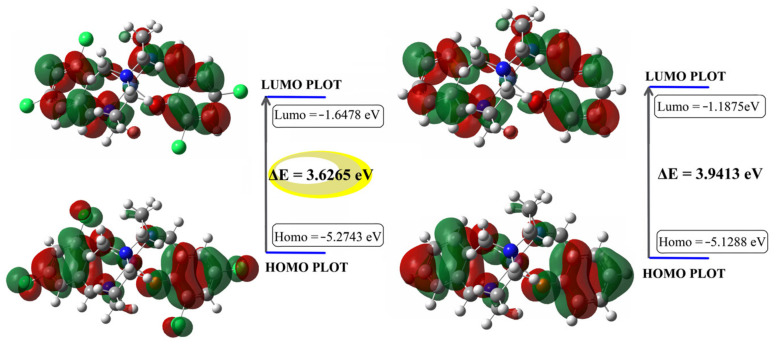
Molecular orbital surfaces for the HOMO and LUMO of [Ni-χ-L] (**a**) and [Ni-L] (**b**) at B3LYP/6-31G(d,p) level.

On the other hand, numerically compared with Ni-L, the LUMO energy level of Ni-χ-L reduced from −1.1875 to −1.6478, indicating the substitution of chlorine atoms also favors the electron-accepting capability of the saldmpn-type complex. On the other hand, the energy gap of Ni-χ-L is 0.3148 eV smaller than that of Ni-L (Figure 8b). From the perspective of energy, a lower bandgap favors the process of importing electrons to LUMO and extracting electrons from HOMO. These results implied the halogenate Ni-χ-L has a higher chemical reactivity than that of Ni-χ-L.

**Figure 9:**
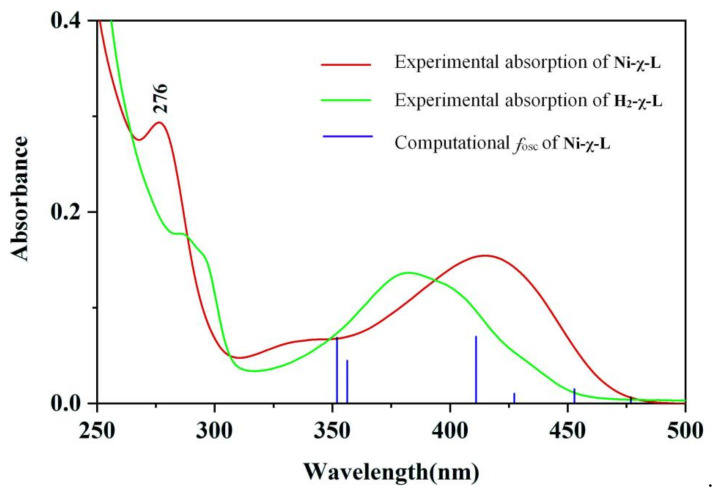
Experimental absorption spectra of Ni-χ-L and H_2_-χ-L, as well as computational data of Ni-χ-L.

The experimental and theoretical studies were performed to better understand the optical properties of the reported compounds. In the UV absorption spectrum, the ligand H_2_-χ-L showed a strong peak absorption peak at 276 nm and a broader peak at 414 nm, which are attributed to the π–π* transition of the benzene rings and n–π* transitions of the C–N bonds, respectively. Whereas the peak at 276 nm nearly disappeared and the peak exhibits blue shifts to 382 nm after H_2_-χ-L was coordinated to the Ni^2+^ center, which can be assigned to the ligand-to-metal charge transfer (LMCT) processes of the newly formed Ni-χ-L (Figure 9).

The B3LYP functional group with a 6-31G(d,p) basis set and implicit polarizable continuum model (PCM) was used to investigate the UV/visible absorption spectra and charge transfer in excited states. In a diluted methanol solution, the dominant broad absorption peak (λ_abs_ = 401 nm) of Ni-χ-L can be predicted by theoretical calculations. Theoretical calculations have shown that the absorption is composed of three different energies at 416 [oscillator strength, f_osc_ = 0.0544; the highest occupied molecular orbital (HOMO)-1→the lowest unoccupied molecular orbital (LUMO) (43%); HOMO-1 → LUMO+1 (42%)]; 356 [oscillator strength, f_osc_ = 0.0334; HOMO-2 → LUMO (97%)]; 355 [oscillator strength, f_osc_ = 0.0536; HOMO-2 → LUMO + 1], respectively, the simulations were generally in agreement with the experimental results.

**Figure 10:**
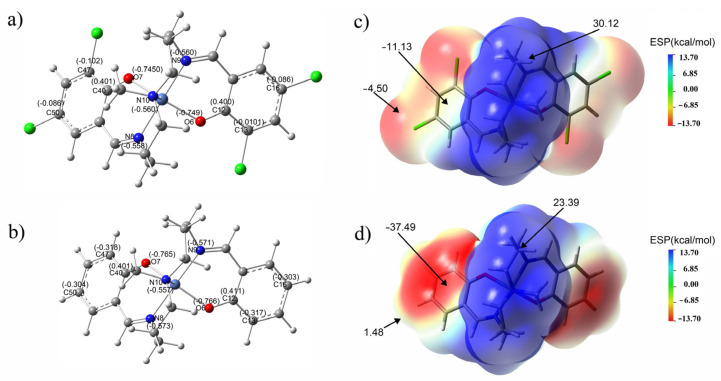
Optimized structures of Ni-χ-L (**a**) and Ni-L (**b**) at B3LYP/6-31G(d,p) level, NBO charges are given on the selected atoms; MEP surfaces of Ni-χ-L (**c**) and Ni-L (**d**), MEP values in Kcal/mol are given in selected points of the surface (MEP for the results are scaled from −13.7 kcal/mol to +13.7 kcal/mol).

The natural bond orbital (NBO) charge distribution of Ni-χ-L is analyzed on the basis of optimized configuration [48,49]. It can be seen that all of the carbon atoms connected to N and O are positive (Figure 10a,b and Figure S8), among which, those bonded to an O atom show the most positive charge (C12:0.400 eV; C46:0.401 eV), which indicates the phenolic oxygen has the strongest electron-withdrawing ability and consequently carries the largest negative charge. Although the electronegativity of Cl is close to that of O, whereas the charge of C atoms connected to Cl is negative (C16: −0.086 eV; C13: −0.0101 eV; C47: −0.102 eV; C50: −0.086 eV;), this result suggests that the conjugation effect of the substituted Cl atoms on saldmpn^2-^ is much stronger than that of the electron-withdrawing effect. Overall, the substituted Cl further increased the conjugation degree of the fluorophore, and enhanced the fluorescence quantum yield of the title compound.

The hydrogen atoms on the carbon atoms attached to the amine nitrogen atoms have the highest positive charges, indicating these hydrogen atoms tend to form hydrogen bond interactions with surrounding atoms. Although nitrogen and oxigen atoms in the complex bear the most negative charge (O6: −0.749 eV; O7: −0.750 eV; N8: −0.560 eV; N9: −0.558 eV; N10: −0.560 eV), these atoms are located at the center of the molecule and enclosed by other atoms, and the steric hindrance impedes the formation of a classic H-bond. Therefore, the neutral molecules can only be connected to each other through multiple weaker interactions. These results also explained, from the NBO aspect, the phenomenon that a smaller driving force can significantly affect the fluorescence intensity in the aggregated state.

Molecular electrostatic potential (MEP) is an important characteristic of a molecular system based on theoretical calculations, which can provide accurate, intuitive and objective viewing of electrostatic properties for the studied molecules [50]. The MEP of Ni-χ-L and Ni-L is shown in Figure 10c,d, from which we can see the most negative regions on the benzene ring for Ni-χ-L and Ni-χ-L are −11.3 kcal/mol and −37.6 kcal/mol, respectively. The substitution of halogen atoms significantly reduces the negative regions of benzene rings; this kind of variation reduced the influence of the solvent, as well as the surrounding molecules on the benzene ring, decreasing the vibrational relaxation effect and can also improve the quantum yield. The above results are consistent with the experimental observations and structural analysis.

## 4. Materials and Methods

### 4.1. Materials

N’,N-bis(3-aminopropyl) methylamine, salicylaldehyde (Aladdin, Shanghai, China), 3,5-dichlorosalicylaldehyde (Aladdin, Shanghai, China), Ni(NO_3_)_2_·6H_2_O (Aladdin, Shanghai, China), HCl (Aladdin, Shanghai, China), N,N-dimethylformamide (DMF) (Aladdin, Shanghai, China), acetonitrile (Aladdin, Shanghai, China), dichloromethane (Aladdin, Shanghai, China), petroleum ether (Aladdin, Shanghai, China), methanol (Aladdin, Shanghai, China), ethanol and CaCl_2_ (Aladdin, Shanghai, China) were analytically pure.

### 4.2. Equipment

UV–vis absorption spectra were recorded by a SHIMADZU UV-2450 (Kyoto, Japan). ^1^H-NMR spectra have been recorded by using a Bruker 300 MHz Digital NMR Spectrometer resonance instrument (AVANCE III 300 MHz) (Billerica, MA, USA). High-resolution mass spectrometry (HRMS) was performed and were analyzed by a Waters Xevo G2-XS QTOF mass spectrometer (Waters Co., Milford, MA, USA). Fluorescence spectra were measured by an Edinburgh FS5 Fluorescence Spectrometer (Livingston, UK). The excitation and emission slits are both set at 0.8 nm with a scanning rate of 600 nm min^–1^. The quantum yields were measured by using a relative approach. Elemental analysis was performed by an Elementar Analysen System GmbH, (Langenselbold, Germany). The single crystal data of the reported compound was collected at a cryogenic temperature (100 K) and measured employing graphite-monochromated MoKα radiation (λ = 0.71073 Å) by a Bruker APEX-II CCD diffractometer (Billerica, MA, USA) equipped with a CCD detector (Bruker); ShelXT were called using the program Olex2. The structures were solved using direct methods, and the remanent atoms were settled from the successive difference Fourier syntheses [51,52]. Hydrogen atoms attached to salicylaldehyde and an amine group were geometrically placed at the calculated positions. All of the non-hydrogen atoms were anisotropically refined.

Fourier Transform Infrared Spectroscopy was recorded at scan range of 400–4000 cm^−1^ and the resolution was taken as 4 cm^−1^; samples were measured by traditional KBr pellet method by a Thermo Scientific Nicolet 6700 DTSG-KBr detector (Waltham, MA, USA).

### 4.3. Synthesis of H_2_-L, H_2_-χ-L, Ni-L and Ni-χ-L

The synthetic route of H_2_-χ-L and H_2_-L is given in Scheme S5a,c. A mix of 3,5-dichlorosalicylaldehyde (0.191 g,1.0 mmol) and N’, N-bis(3-aminopropyl) (0.0125 M) were dissolved in EtOH (40 mL) and stirred at 60 °C for 3 h. After cooling the mixture, CH_2_Cl_2_ (5 mL) and petroleum ether (40 mL) were added to the reaction mixture, then anhydrous CaCl_2_ (5 g) was added and left to stand for five hours at room temperature. The filtrate was rotary evaporated at 60 °C for one hour, then a yellow amorphous powder was obtained, namely H_2_-χ-L. Yield, 76.2%. Anal. Calcd. for C_21_H_23_Cl_4_N_3_O_2_: C, 51.34; H, 4.72; N, 8.55. Found: C, 51.28; H, 4.77; N, 8.60. HRMS calcd. 491.2371, found 491.2679. FT-IR of H_2_-χ-L: 456(ν-Cl); 1065(ν_C-O_); 1197~1296(ν_-OH_);1424(ν_-CH3_); 1660(ν_C=N_); 2823~2935 (ν_C-H_). ^1^H NMR (300 MHz, CDCl_3_-d_1_, δ): 9.58 (s, 2H, OH), 8.56 (s, 2H, CH = N), 7.51 (dd, J = 15.0, 6.5 Hz, 4H, Ar-H), 3.71 (dd, J = 5.0, 4.9 Hz, 4H, CH_2_-N), 2.46 (dd, J = 5.2, 5.1 Hz, 4H, CH_2_-N), 2.18 (s, 3H, CH_3_), 1.75–1.37 (m, 4H, CH_2_-N). ^13^C NMR (101 MHz, Chloroform-d) δ 157.5, 157.9, 134.1, 128.7, 128.4, 127.4, 126.9, 59.5, 57.0, 47.2, 29.5.

H_2_-L was synthesized using the same procedure with H_2_-χ-L, except that salicylaldehyde (0.122 g, 1 mmol) was used instead of 3,5-dichlorosalicylaldehyde (0.191 g, 1 mmol). Yield, 83.4%. Anal. Calcd. for C_21_H_27_N_3_O_2_: C, 71.36; H, 7.70; N, 11.89. Found: C, 71.26; H, 7.77; N, 11.82. HRMS calcd. 353.4581 found 353.4996. FT-IR of H_2_-L: 1048(ν_C-O_); 1205~1332(ν_-OH_); 1446(ν_-CH3_); 1650(ν_C=N_); 2843~2942 (ν_C-H_). ^1^H NMR of [H_2_-L] (300 MHz, Chloroform-d, δ): 11.11 (s, 2H, OH), 8.56 (s, 2H, CH=N), 7.65 (dd, J = 6.4, 2.1 Hz, 2H, Ar-H), 7.32-7.29 (m, 2H, Ar-H), 7.16-7.12 (m, 2H, Ar-H), 6.93 (dd, J = 5.8, 2.4 Hz,2H, Ar-H), 3.71 (dd, J = 5.1, 5.0 Hz, 4H, CH_2_-N), 2.46 (dd, J = 5.2, 5.2 Hz, 4H, CH_2_-N), 2.18 (s, 3H, CH_3_), 1.75-1.37 (m, 4H, CH2-N). ^13^C NMR of [H_2_-L] (101 MHz, Chloroform-d) δ 157.9, 157.5, 134.1, 128.7, 128.4, 127.4, 126.9, 59.5, 57.0, 47.2, 29.5.

The synthetic route of Ni-L and Ni-χ-L is shown in Figure S5b,d. Ni(NO_3_)_2_·6H_2_O (0.5 mmol) was added to twenty milliliters of H_2_-χ-L ethanol solution (0.025 M) at room temperature while stirring for 1 h. The solution was filtered and the filtrate was slowly volatilized at room temperature. Brown block crystals were obtained after a week. Yield: 51.3%. Anal. Calcd. for C_21_H_21_Cl_4_N_3_O_2_Ni: C:46.0; H:3.86; N:7.67; Found:C:45.6; H:3.92; N:7.69. HRMS calcd. 547.9147 found 547.9784. FT-IR of Ni-χ-L (KBr, 400–4000 cm^−1^): 431(ν_-Cl_); 879(ν_Ni-O_,ν_Ni-N_); 1045(ν_C-O_); 1380(ν_-CH3_); 1650(ν_C=N_); 2888–2974(ν_C-H_); 3325(ν_O-H_); Ni-L was synthesized using the same procedure with Ni-χ-L, except that salicylaldehyde (0.122 g, 1 mmol) was used instead of 3,5-dichlorosalicylaldehyde (0.191 g, 1 mmol). yield: 51.3%. Anal. Calcd. for C_21_H_27_N_3_O_2_: C:46.0; H:3.86; N:7.67; Found:C:45.6; H:3.92; N:7.69. HRMS calcd. 353.4581, found 353.4923. FT-IR of Ni-L (KBr, cm^−1^): 879(ν_Ni-O_,v_Ni-N_); 1045(ν_C-O_); 1380(ν_-CH3_); 1641(ν_C=N_); 2893–2974(ν_C-H_); 3319(ν_O-H_).

[CCDC 2082176 contains the supplementary crystallographic data for this paper. These data can be obtained free of charge from The Cambridge Crystallographic Data Centre via www.ccdc.cam.ac.uk/data_request/cif (accessed on 7 May 2021)].

### 4.4. Association Constant and Detection Limit

Since the ratio of ligand to metal was confirmed to be 1:1, the absorbance at 334 and 384 nm for Ni-χ-L and Ni-L and the stability constant of the compounds were determined using the UV titration curve and fitted with following equation:

(1) $$1/\left(A-A_{0}\right)=1/\left[Ni^{2+}\right]K\left(\varepsilon_{PB_{n}}-\varepsilon_{P}\right)C_{P}^{0}]+1/\left[\left(\varepsilon_{PB_{n}}-\varepsilon_{P}\right)C_{P}^{0}\right]$$

In the same way, the absorbance at 334 nm and 384 nm were chosen and the following simple formula: LOD = 3 σ⁄m, was used to calculate the lowest detection limit of Ni-χ-L and Ni-L (Where “σ” is the standard deviation of the absorbance at 334 or 384 nm of blank measurement, “m” is the slope of the linear fitting curve).

### 4.5. pH Titration

The pH sensitivity of Ni-χ-L was studied by using HCl or NaOH in an ethanol/water mixture (20:1, *v*/*v*). The ethanol solutions of HCl and NaOH were added to the solution of Ni-χ-L (c= 400 μM), respectively. The fluorescence emission spectra of the solutions were measured at every pH unit (λex = 360 nm).

### 4.6. Quantum Yield (QYs) Measurement

Quinine sulfate was chosen as a reference fluorophore of known quantum yield (QY = 54% in 0.1 M H_2_SO_4_). Then, the QY of the samples were calculated according to the following equation:

(2) $$\Phi =\Phi^{\prime }\times \left(A^{\prime }/I^{\prime }\right)\left(I/A\right)(n^{2}/n{’}^{2})$$

where *ϕ* is the testing sample’s QY is the integrated emission intensity of the testing sample, *n* is the refractive index (1.36 for ethanol), and *A* is the integral area of the fluorescence emission spectrum. In order to obtain more reliable results, the concentration was adjusted to maintain the optical absorption values at 360 nm, in the range from 0 to 0.1. The fluorescence emission spectrum was measured and the fluorescence intensity was integrated. QYs are determined by comparison of the integral fluorescence intensity with absorbance curves (refractive index n must to be taken into account).

### 4.7. The pH Response of Different Metals

The buffer solution of different metal (Mn^2+^, Fe^3+^, Co^2+^, Ni^2+^, Cu^2+^, Ag^+^, Al^3+^, Bi^3+^, Ca^2+^, Cr^2+^, K^+^, Li^+^, Mg^2+^, Na^+^ and Sr^2+^) complexes with the same concentration were prepared in the ethanol solution of pH 5 and 6. The fluorescence emission spectra of the solutions were measured. (λex = 360 nm).

### 4.8. Stability and Reversibility

Ethanol/water solutions (10, *v*/*v*) (c = 400 μM) of Ni-χ-L and Ni-L were prepared. The pH value was regulated between pH 5 and 6 by NaOH (1 × 10^5^ μM) and HCl (1 × 10^5^ μM) aqueous solutions. The fluorescence intensity of the solutions was measured each time pH reached 5 and 6. In addition, samples of the same concentration (c = 400 μM) of pH 5 and 6 were exposed to sunlight, and their fluorescence intensity measurements were taken at regular intervals.

### 4.9. Computational Methods

The calculation accuracy and time-consuming are substantially affected by the calculation method and basis set. Previous studies indicated that the B3LYP method can achieve satisfactory results in explaining the reaction mechanism and reactivity of the Schiff base complexes. Therefore, in this work, the DFT method B3LYP with a standard 6–31G(d,p) basis set was used for the geometry optimizations [46,47]. By comparing the optimized geometrical configurations and the structure, determined by X-ray diffraction, the relationship between molecular orbitals and reactivity was explored. Meanwhile the natural bond orbital (NBO) was also analyzed in this work, which was determined by the keyword POP = NBO [48,49]. All calculations were performed using the default Gaussian16W package [53].

## 5. Conclusions

In summary, a new Schiff base type colorimetric and fluorescent pH probe has been synthesized. The probe showed a good pH response with fluorescence signal significantly enhanced 15 times as pH increased from 5.1 to 6.0. In addition, the probe has good linearity, stability and reversibility; moreover, the differences in the signal intensities between different pH values can effectively avoid the adverse effects of self-luminescence and ambient light.

To achieve a deeper understanding of the special fluorescence behavior of the reported compound, we introduced the non-halogenated isomer for comparison in terms of structural analysis, optical properties, as well as theoretical calculation, etc. The influence of halogenated groups on the fluorescence properties of fluorophores and the unusual signal strength were also analyzed and discussed.

We believe that, to some extent, the present work can provide new guidance for the design and synthesis of new probes and increase the signal intensity of reported fluorophores. Future work will focus on broadening the detection range and exploring a feasible way to integrate the biological detection and physiological regulation of Schiff base probes.

## Data Availability

Not applicable.

## Supplementary Materials

The following are available online at https://www.mdpi.com/article/10.3390/ijms23116259/s1.

## Abbreviations

H_2_-χ-L	3:5-Cl-saldmpn = N, N’-( 3,3′-dipropyhnethylamine) bis (3,5-chlorosalicylidene)
Ni-χ-L	Ni^II^(3,5-Cl-saldmpn)
H_2_-L	saldmpn = N, N’-( 3,3′-dipropyhnethylamine) bis (salicylideneiminato)
Ni-L	Ni^II^ (saldmpn)
