# Supplementary material for: Rigid Schiff Base Complex Supermolecular Aggregates as a High-Performance pH Probe: Study on the Enhancement of the Aggregation-Caused Quenching (ACQ) Effect via the Substitution of Halogen Atoms
Source: Int J Mol Sci. 2022 Jun 2;23(11):6259. doi: 10.3390/ijms23116259 (PMC9181572; doi:10.3390/ijms23116259)
Supplement: Supplementary file 1 [file ijms-23-06259-s001.zip › ijms-1733128-SI.pdf]

Electronic Supplementary Information

Rigid Schiff Base Complex Supermolecular Aggregates as a High-Performance pH Probe: Study on the Enhancement of the Aggregation-Caused Quenching (ACQ) Effect via the Substitution of Halogen Atoms

Tianyu Li [1,2], Haijun Pang[4], Qiong Wu [1,3*], Meifen Huang [1], Jiajun Xu [1], Liping Zheng [1,2], Yongfeng Qiao [1*], Baoling Wang [1,3*]

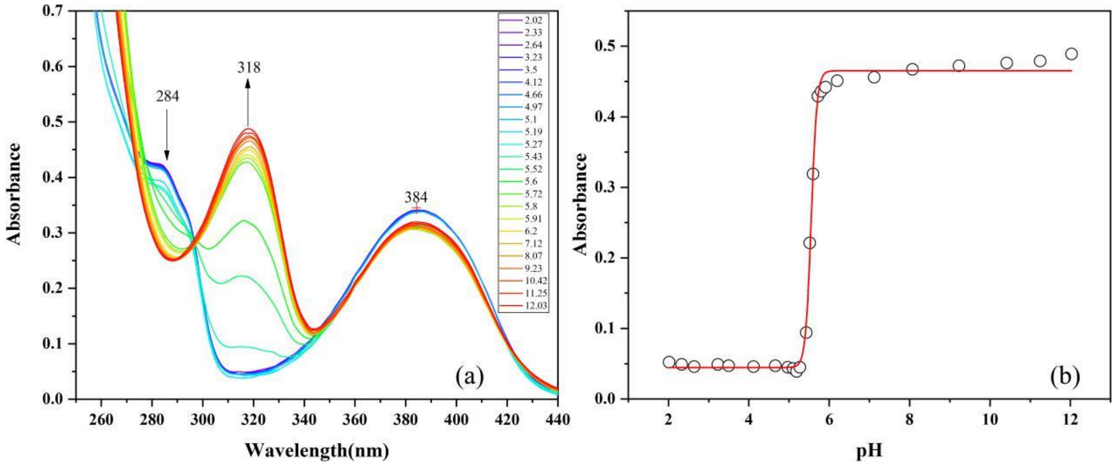

Figure S1. a) UV/Vis spectral changes of **1** ($0.4*10^{-4}$ M) by the addition of NaOH (0.1 M) and HCl (0.1 M); b) relationship between fluorescence intensities and pH (λex = 318 nm).

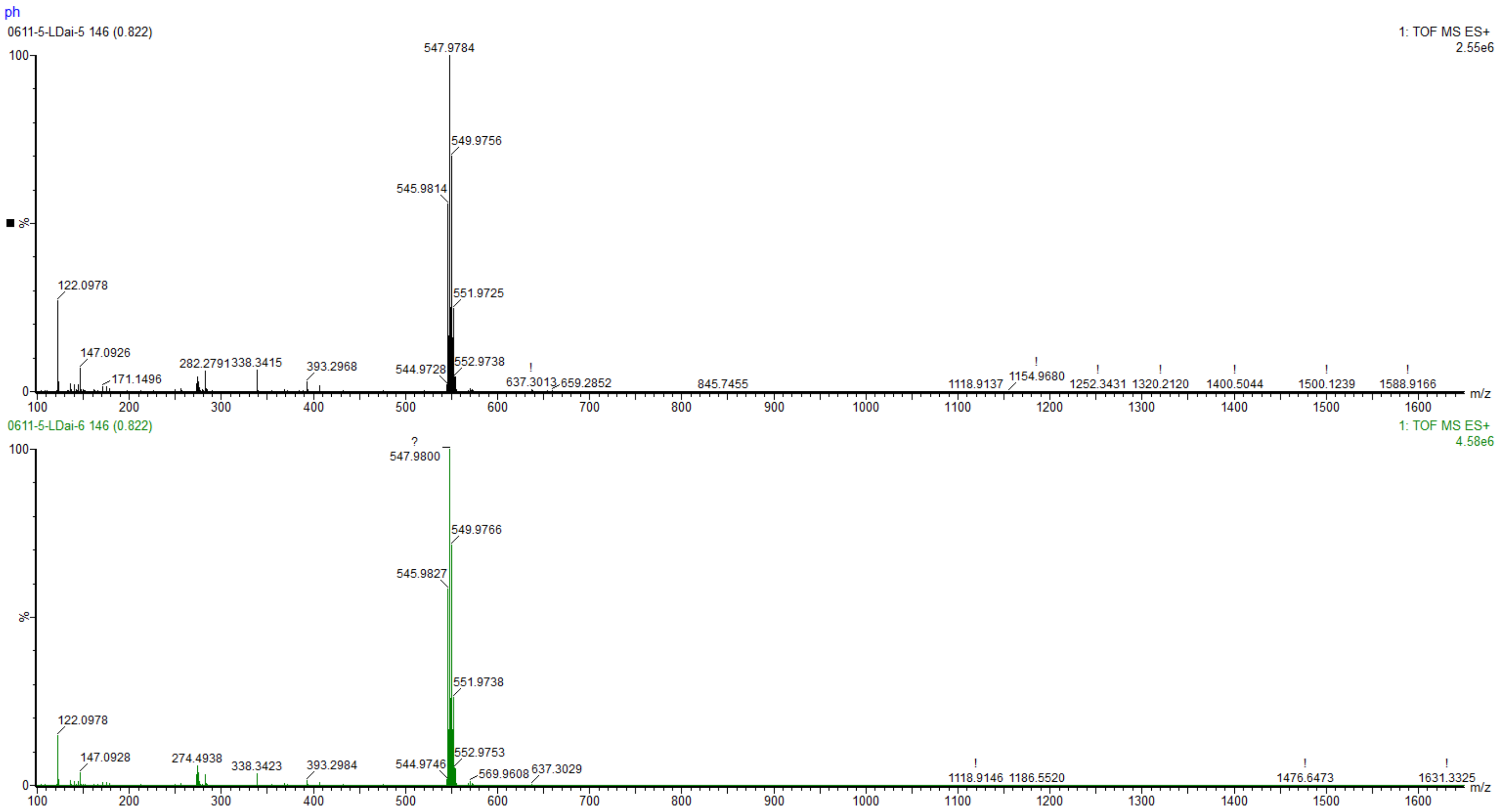

Figure S2. The raw MS spectra of Ni-χ-L at pH = 5(up) and 6(down).

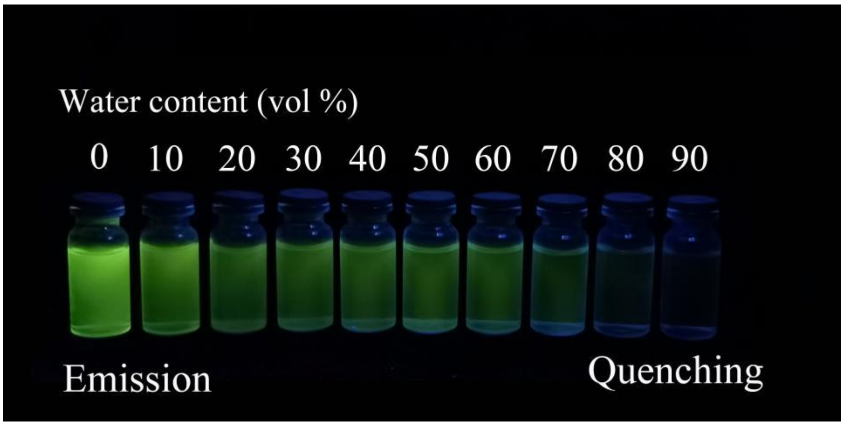

Figure S3. The photographs of [Ni-χ-L]$_3$ (**1**) in water/EtOH media with water fractions of 0-90% (excited at 365 nm).

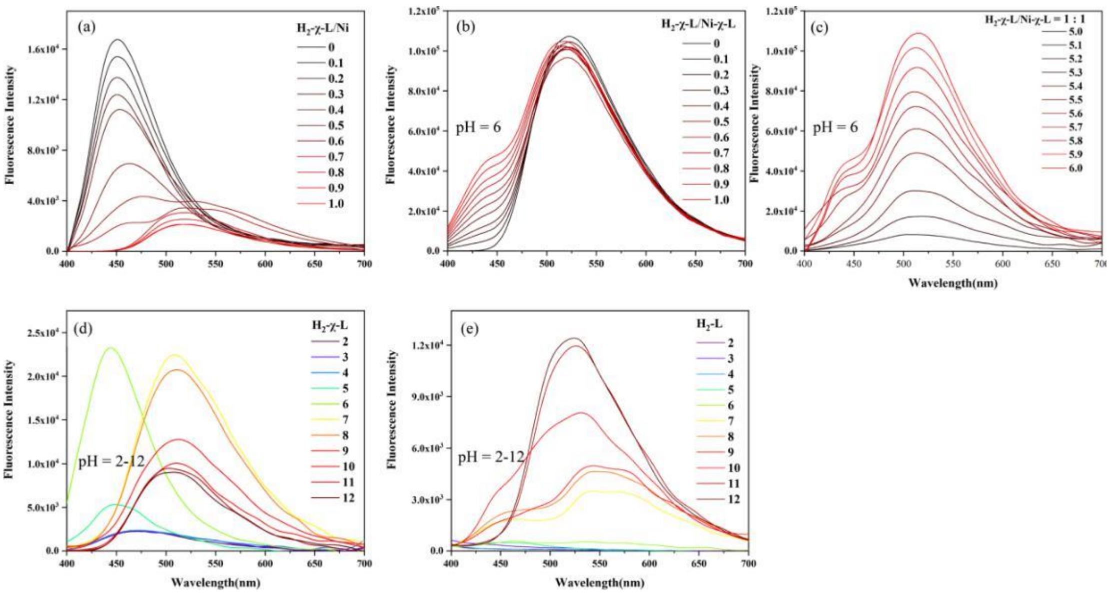

Figure S4. Changes in emission spectra of a) H_2-χ-L (400 μM) upon the addition of Ni^{2+} (λ_{ex} = 360 nm) at room temperature. [Ni^{2+}] = 0, 40, 80, 120, 160, 200, 240, 280, 320, 360, 400, 440 μM); b) Ni-χ-L at pH= 6 (400 μM) with the addition of H_2-χ-L (λex = 360 nm) at room temperature. H_2-χ-L = 0, 40, 80, 120, 160, 200, 240, 280, 320, 360, 400 μM); c) the pH titration of equivalent mol of Ni-χ-L complex and free ligand, the pH adjusted by aqueous solution of NaOH (1×10^{5}μM) and HCl (1×10^{5}μM) (λ_{ex} = 360 nm) at room temperature; d) and e) the pH titration of H_2-χ-L and H_2-L with the pH change from 2 to 12, the pH adjusted by aqueous solution of NaOH (1×10^{5}μM) and HCl (1×10^{5}μM) (λ_{ex} = 360 nm) at room temperature.

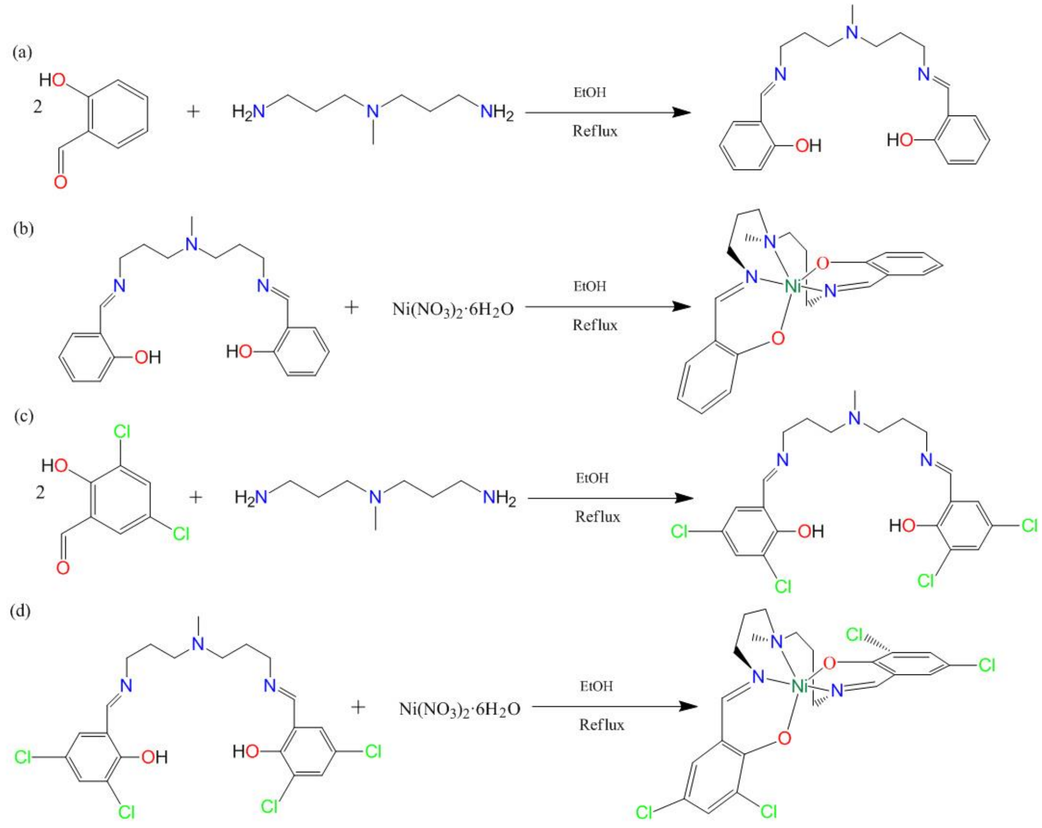

Figure S5: Synthetic routes of Ni-χ-L

Table S1. Crystallographic data and structure refinement for compound **1**

Empirical formula	$C_{63}H_{63}Ni_3Cl_{12}N_9O_6$
Formula weight	1643.66
Temperature/K	150(2)
Crystal system	monoclinic
Space group	$P2_1/c$
a/Å	10.5453(2)
b/Å	36.2658(9)
c/Å	18.2268(5)
β/°	100.6040(10)
Volume/Å^3	6851.5(3)
Z	4
ρ_{calc}g/cm^3	1.526
μ/mm^{-1}	1.339
F(000)	3097.0
Crystal size/mm^3	0.16 ×0.16 × 0.12
Radiation	MoKα (λ = 0.71073)
2Θ range for data collection/°	4.064 to 52.758
Index ranges	$-13 \leq h \leq 13$, $-45 \leq k \leq 45$, $-22 \leq l \leq 22$
Reflections collected	97212
Independent reflections	14008 [R_{int} = 0.0535, R_{sigma} = 0.0294]
Data/restraints/parameters	14008/0/841
Goodness-of-fit on F^2	1.042
Final R indexes [I>=2σ (I)]	R1 = 0.0549, wR2 = 0.1494
Final R indexes [all data]	R1 = 0.0698, wR2 = 0.1620
Largest diff. peak/hole / e Å^{-3}	2.40/-1.19

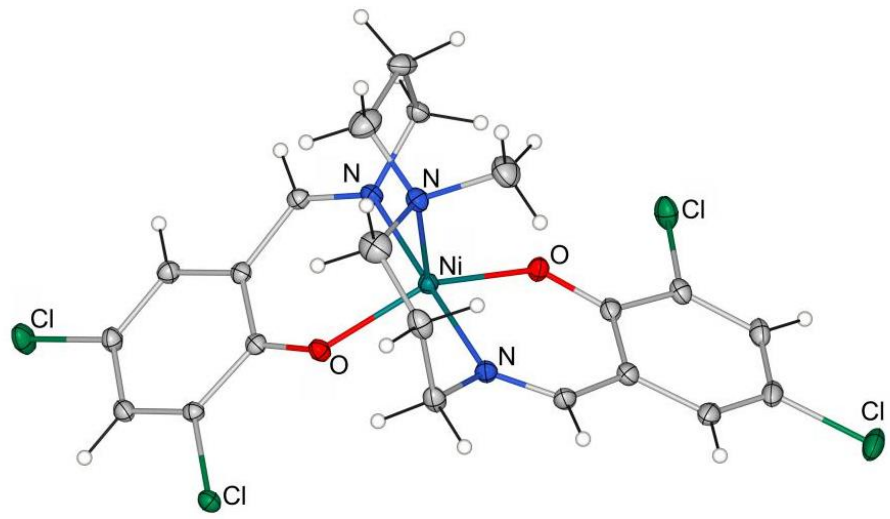

Figure S6. The monomeric structure of 1, showing the selected atom type labeling. Displacement ellipsoids are drawn at the 30% probability level.

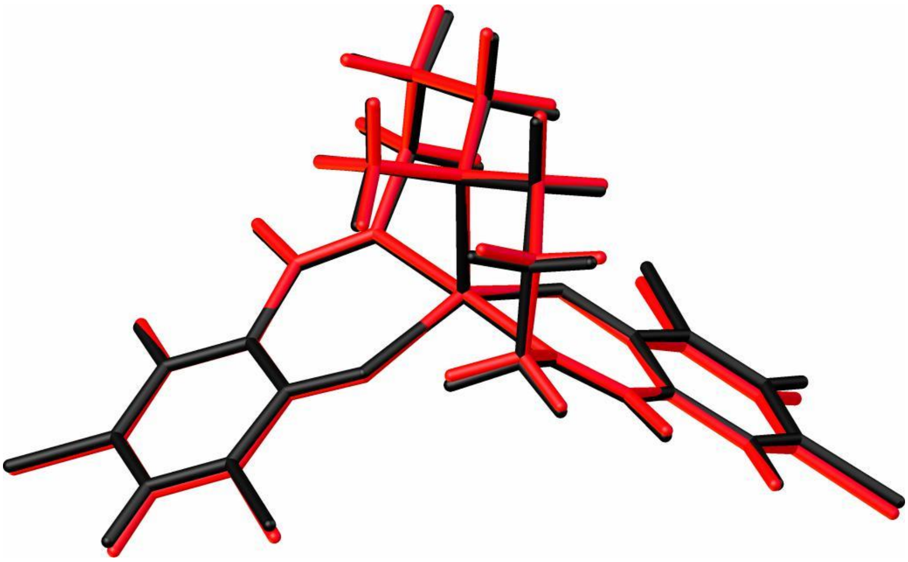

Figure S7 A molecular fit of the experimental and optimized structures of **1** shown in black and red, respectively.

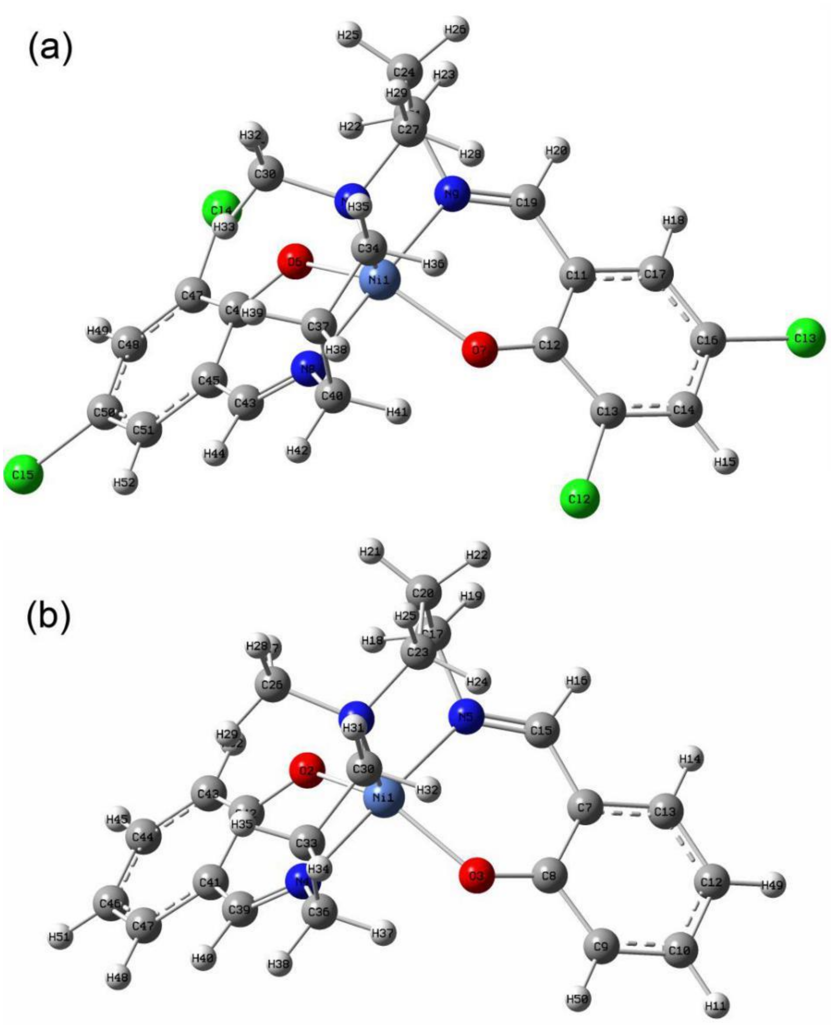

Figure S8. Optimized structure and the atomic atomic number of [Ni-χ-L] (a) and [Ni-L] (b).
